# Silencing NOB1 enhances doxorubicin antitumor activity of the papillary thyroid carcinoma *in vitro* and *in vivo*

**DOI:** 10.3892/or.2022.8297

**Published:** 2022-03-01

**Authors:** Jia Liu, Bing-Fei Dong, Pei-Song Wang, Pei-You Ren, Shuai Xue, Xiao-Nan Zhang, Zhe Han, Guang Chen

Oncol Rep 33: 1551–1559, 2015; DOI: 10.3892/or.2015.3730

Following the publication of this paper, it was drawn to the Editors’ attention by a concerned reader that certain of the western blotting assay data shown in Figs. 2 and 5, and the tumour images shown in Fig. 6A, were strikingly similar to data appearing in different form in other articles by different authors. Owing to the fact that the contentious data in the above article had already been published elsewhere, or were already under consideration for publication, prior to its submission to *Oncology Reports*, the Editor has decided that this paper should be retracted from the Journal. After having been in contact with the authors, they agreed with the decision to retract the paper. The Editor apologizes to the readership for any inconvenience caused.

